# A modified frailty index to identify high-risk groups for amyotrophic lateral sclerosis

**DOI:** 10.3389/fneur.2026.1879866

**Published:** 2026-07-15

**Authors:** Xuecai Gao, Sheng Chen, Sarwara Mahabub, Baokun Cao, Jiahe Deng, Weimin Ye, Haomin Yang, Zhangyu Zou

**Affiliations:** 1 Department of Epidemiology and Health Statistics, School of Public Health, Fujian Medical University, Fuzhou, China; 2 Department of Neurology, Fujian Medical University Union Hospital, Fuzhou, China; 3 Department of Medical Epidemiology and Biostatistics, Karolinska Institutet, Stockholm, Sweden

**Keywords:** ALS, frailty, modified frailty index, risk stratification tools, UK Biobank

## Abstract

**Background:**

Amyotrophic lateral sclerosis (ALS) is a motor neuron disease characterized by progressive muscle weakness and poor prognosis, which requires early detection to optimize therapeutic outcomes. This study aims to develop a risk stratification tools for ALS and to assist in identifying high-risk groups.

**Methods:**

A prospective cohort study was conducted using the UK Biobank (500,033 participants), which were split into training sets and validation sets. We calculated the frailty index (FI) and modified frailty index (MFI) for the participants and estimated their association with ALS. Finally, two risk stratification tools were constructed and the time-dependent ROC curve was utilized to evaluate the discriminatory performance of each model.

**Results:**

Among the 49 deficits in FI, we identified five deficits that were significantly associated with ALS, including falls, whole-body pain, long-standing illness, disability or infirmity, self-rated health and tiredness or lethargy in last 2 weeks, which together constructed the MFI. Both the FI and MFI were associated with a higher risk of ALS (HR_FI_ = 4.58, 95% CI = 1.31–16.07, HR_MFI_ = 4.59, 95% CI = 2.79–7.53). Finally, a combination of MFI, gender, age and BMI demonstrated the best discriminative ability. Specifically, on the validation set, it achieved a C-index of 0.696.

**Conclusion:**

By focusing on deficits associated with ALS, the MFI may improve the ability to identify individuals at elevated risk of the disease. It could therefore serve as a valuable screening tool for risk stratification in the general population.

## Introduction

Amyotrophic lateral sclerosis (ALS) is a progressive and fatal motor neuron disease characterized by degeneration of upper and lower motor neurons, which leads to muscle atrophy in the bulbar and limbs (1). The prognosis for patients with ALS is poor, with a typical survival of only 3 to 5 years (2, 3). Due to the lack of diagnostic biomarkers for ALS, the delayed diagnosis of ALS patients is usually between 10 to 16 months (4). Notably, early diagnosis can improve the therapeutic effect or ensure timely drug administration within the treatment window (5), the development of effective early screening tool to identify high-risk individuals in the general population is of critical importance for patients with ALS.

However, most studies have focused on the prognosis or disease progression of ALS patients (6–9), while few researchers have developed risk stratification tools that can be used to assess the risk of ALS. The ALS risk prediction models based on biomarkers in plasma or cerebrospinal fluid (CSF) have demonstrated high sensitivity and specificity (10, 11). However, the requirement for various blood or CSF biomarkers significantly limits the applicability of these models in the general population, thereby hindering the identification of high-risk individuals for ALS.

The Frailty Index (FI) serves as a key indicator for evaluating an individual’s physiological aging status, and it has been closely linked to the incidence of aging-related diseases (12, 13). ALS is more common in the elderly over 60 years old, and regarded as a neurodegenerative disease related to frailty (14). To further improve the predictive ability of FI, a modified FI (MFI) has demonstrated significant value to predict outcomes after surgery (15–18). However, no study has systemically evaluated the role of FI or MFI in screening for ALS risk.

This study aims to develop an early screening tool to identify individuals at elevated risk of ALS in the general population. In addition, two models were constructed by combining FI, MFI, gender, age and BMI, and their discriminative abilities were compared.

## Methods

### Study population

This cohort study included approximately 500,000 participants in the UK Biobank (UKB), with age between 40 and 69 years, and recruited across the UK from 2006 to 2010 (19, 20). The UKB collected various types of phenotypic information and biological samples from the participants, mainly including various types of demographic information, lifestyle characteristics, blood samples, and body and brain images (21). The UKB participants were followed up from their date of enrollment until the date of diagnosis of ALS, date of death, loss of follow up or until the end of study (31 December 2021), whichever came first. Information on ALS diagnosis was obtained by using unique personal identification numbers to link the cohort to the National Health Service (NHS) Digital for England and Wales, and National Records of Scotland, NHS Central Register for Scotland. The ICD-10 code G12.2 was used to identify ALS diagnosis. The date of death was retrieved from death certificates held by the NHS Information Center and the NHS Central Register. Participants with ALS before enrollment were excluded from the analysis. The UKB was approved by The National Information Governance Board for Health and Social Care and the NHS North West Multicentre Research Ethics Committee (Ref: 11/NW/0382, 17 June 2011), and participants provided written informed consent. This research was conducted using the UK Biobank Resource under Application 61,083.

### Calculation of the frailty index

The FI of UKB participants was calculated in accordance with the standard procedure (22), which was the same as the work of Williams et al. (12). Deficits were assessed using a variety of physical or mental health status, including symptoms, diagnosed diseases, and disabilities. A total of 49 health deficit indicators were utilized, specifically including clinical phenotypes of the cardiovascular system, head and facial structures, tumors, respiratory system, endocrine metabolism, digestive system, multisite pain, and mental health. In calculating the score for each deficit, continuous variables were combined with categorical or binary variables to assign a value to each trait based on the severity of the deficit (zero meaning a deficit is absent, and one meaning the deficit is at its most severe). Participants with more than 10 missing items in the calculation of the FI were excluded from the analysis, leaving 500,033 participants ultimately included in this study, of whom 628 were reported to have ALS during the follow-up.

### Statistical analysis

In this study, the dataset was randomly divided into a training set (*n* = 350,024) and a validation set (*n* = 150,009) using a 7:3 allocation ratio. Chi-squared tests were conducted to compare the baseline characteristics of the participants (Supplementary Table 1). To identify health deficits that are associated with the ALS, we first performed univariate Cox regression analyses on each of the 49 deficits included in the FI. Deficits that were significantly associated with incident ALS at a false discovery rate (FDR) < 0.05 were selected. Using these ALS associated deficits, we then constructed a MFI for each participant. All selected deficits were assigned equal weight, and the MFI was calculated as the sum of the deficits present divided by the total number of selected deficits, yielding a score ranging from 0 to 1.

We calculated the Variance Inflation Factor (VIF) for each variable to assess potential multicollinearity among the deficits included in the MFI. The associations of FI and MFI with ALS were then examined using a Cox regression model, adjusting for baseline characteristics (including gender, age, ethnicity, BMI, smoking, alcohol frequency and Townsend deprivation index). The analyses were further stratified by baseline characteristics, and their interactions were tested by adding an interaction term.

To assess potential reporting bias, we excluded participants with poor baseline self-rated health and re-estimated the association between the MFI and ALS. To evaluate reverse causation, we sequentially excluded incident ALS cases diagnosed within 3, 5, 8, and 10 years of follow-up, re-estimating the HR each time. Furthermore, the association of MFI with ALS over time was examined using a flexible parametric model (FPM).

To address missing data in the database, we applied Multiple Imputation by Chained Equations (MICE). Imputation was performed for variables among the 49 deficits that contained missing values, with outcome variables and covariates included as predictors to improve imputation accuracy. Predictive models were specified according to variable type, with logistic regression used for binary variables. A total of five imputed datasets were generated, each with 20 iterations. Subsequent statistical analyses were conducted independently on each imputed dataset, and the results were combined using Rubin’s rules to obtain robust effect estimates and standard errors.

Firstly, multivariate Cox regression with backward selection was used to construct a model based on FI and other demographic and lifestyle factors (Model A). Meanwhile, model B was constructed based on MFI and other baseline characteristics. For each model, the C-index was calculated. In addition, we used the time-dependent receiver operating characteristic (ROC) curve to assess the discriminatory performance of each model within a specific time period. Finally, we evaluated the performance of model A and model B in the validation set.

All statistical analyses were conducted in STATA version 17.0, while the forest plots and ROC curves were created using the “forestploter” and “timeROC” package in R version 4.4.2.

## Results

### Baseline characteristics

The baseline data of the UK Biobank participants are presented in Supplementary Table 1. There were statistically significant differences in gender, age and smoking status (*p* < 0.05) between ALS patients and non-patients in the training set, while the gender, age and ethnicity showed statistical differences in the validation set, with no differences in other characteristics.

### Modified frailty index for ALS

Among the 49 deficits, five of them were significantly associated with ALS (FDR < 0.05, Table 1; Supplementary Table 2), including falls, whole-body pain, long-standing illness, disability or infirmity, self-rated health and tiredness or lethargy in last 2 weeks. Consequently, these deficits were summed up as the MFI. To address potential collinearity among variables, we calculated the variance inflation factor (VIF) for each variable. The VIF values ranged from 1.05 to 1.43, well below the conventional threshold of 5 (Supplementary Figure 1).

**Table 1:** The associations between health deficits in the modified frailty index (MFI) and ALS.

Health deficits	HR (95%CI)	*p*-valve	Adjusted *p*-valve
Falls	2.13 (1.62–2.81)	<0.001	0.002
Whole-body pain	2.64 (1.64–4.25)	<0.001	0.002
Long-standing illness, disability or infirmity	1.42 (1.17–1.73)	0.001	0.012
Self-rated health	2.00 (1.31–1.73)	0.001	0.012
Tiredness or lethargy	1.67(1.21–2.31)	0.002	0.020

The model was adjusted for age, gender, ethnicity, BMI, smoking, alcohol frequency and Townsend deprivation index.

**Figure 1:**
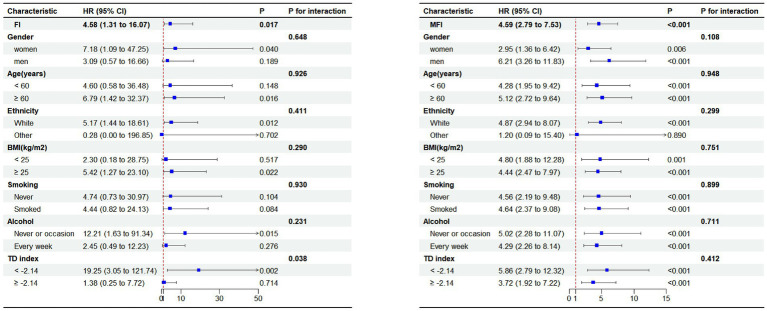
Forest plot of the effect of FI and MFI on ALS and stratified by baseline characteristics. The models are adjusted by baseline characteristics (including gender, age, ethnicity, BMI, smoking, alcohol frequency and Townsend deprivation index). Caution is warranted when interpreting estimates in this stratum due to the limited number of events.

The FI and MFI was associated with a higher risk of ALS (HR_FI_ = 4.58, 95% CI = 1.31 ~ 16.07, HR_MFI_ = 4.59, 95% CI = 2.79 ~ 7.53). In the stratified analysis, higher FI increased the risk of ALS among the participants with a low Townsend Deprivation Index with an HR of 19.25 (95%CI = 3.05 ~ 121.74), and the effect in the participants with a high Townsend Deprivation Index was 1.38 (95%CI = 0.25 ~ 7.72). There was no interaction between FI, MFI and other baseline characteristics (Figure 1).

After excluding participants who self-rated health as “Poor,” the association between the MFI and ALS remained statistically significant (HR = 3.81, 95% CI = 1.92 ~ 7.54). We sequentially excluded incident ALS cases diagnosed within 3, 5, 8, and 10 years of follow-up. The association between the MFI and ALS risk attenuated gradually but remained significant up to 8 years (3 years: HR = 3.42, 95% CI 1.98 ~ 5.93; 5 years: HR = 2.94, 95% CI 1.63 ~ 5.30; 8 years: HR = 2.38, 95% CI 1.13 ~ 4.98). At 10 years, the association was no longer statistically significant (HR = 1.72, 95% CI 0.66 ~ 4.49, *p* = 0.269).

The flexible parametric model showed that the HR of MFI was the highest at the beginning of the follow-up period, but had a continuous downward trend as the follow-up time extends. The effect was no longer significant approximately after a 10-year follow-up (Supplementary Figure 2).

### Comparing models

**Figure 2:**
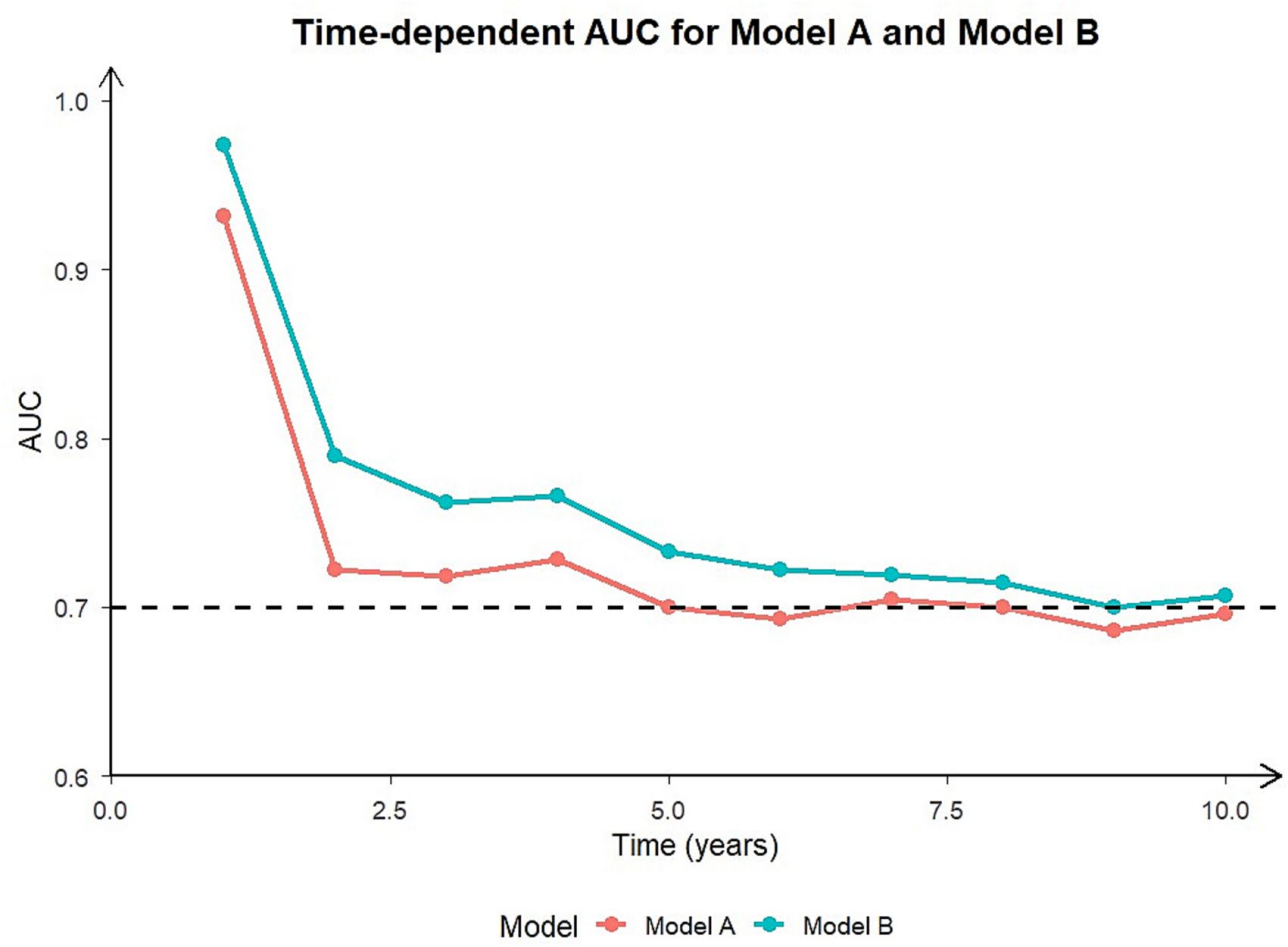
AUC values from time-dependent roc analysis of different als risk stratification tools.

First, FI based multivariate Cox regression model with backward stepwise method was used, with FI, gender and age finally included in the model (Model A, AUC in the validation set: 0.688, 95% CI = 0.654 ~ 0.722). The same statistical method was applied to generate MFI based model, while MFI, gender, age and BMI were left in the model (Model B, AUC in the validation set: 0.696, 95% CI = 0.663 ~ 0.730). However, no statistically significant difference was observed between model A and model B in the validation set (*p* = 0.218, Supplementary Table 3). Finally, the time-dependent ROC analysis demonstrated that model B maintained AUC values consistently above 0.7 over a period of 1 to 10 years (Figure 2).

### Screening ability for high-risk populations

**Figure 3:**
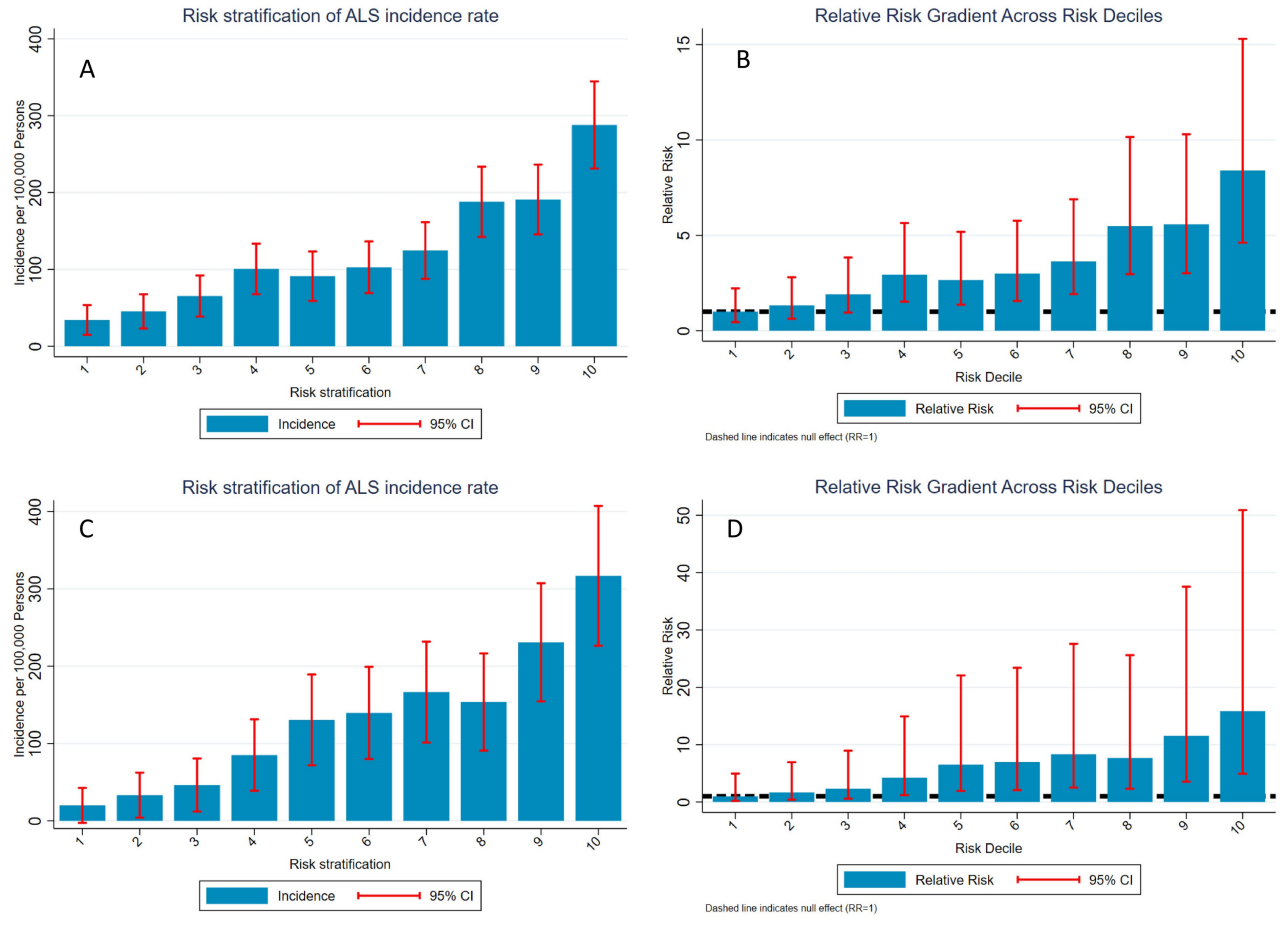
ALS incidence trend plot and relative risk trend plot based on Model B. **(A)** ALS incidence trend plot in training set. **(B)** ALS relative risk trend plot in training set. **(C)** ALS incidence trend plot in validation set. **(D)** ALS relative risk trend plot in validation set.

We employed model B to compute risk scores for all participants, and then stratified the study population into 10 equal groups based on these scores. For each risk stratum, we calculated the cumulative incidence and relative risk (RR). Results showed that in the training set, the highest-risk stratum had a cumulative incidence of 287.95 per 100,000 individuals, with an RR 8.41 times that of the lowest-risk stratum. In the validation set, the highest-risk stratum exhibited a cumulative incidence of 316.84 per 100,000 individuals, and its RR was 15.84 times that of the lowest-risk stratum (Figure 3).

## Discussion

In this study, two risk stratification models were constructed based on the multidimensional data resources of the UK Biobank, which integrates baseline demographic information and participants’ clinical phenotypes. Although the difference in C-index between the two models did not reach statistical significance, time-dependent ROC analysis revealed that model B consistently outperformed model A in terms of AUC values. This finding implies that while summary measures such as the C-index may suggest comparable overall discrimination, model B exhibits a sustained advantage in discriminatory performance over time. Given its validated performance, model B may serve as a useful screening tool for identifying high-risk groups of ALS.

Among the five deficits in the MFI, the health consequences triggered by fall events in the elderly population are clinically significant, leading to serious complications such as hip fracture and traumatic brain injury. Notably, studies in recent years have suggested a possible association between trauma and the risk of developing ALS (23). Similarly, pain is common in ALS patients (24, 25), while few studies have focused on the association between pain and the risk of ALS. Because of the insidious onset of ALS and its confusion with other neuromuscular disorders, potential ALS patients with generalized pain symptoms are highly likely to be an early symptom of ALS.

Long-standing illness or infirmity, declining self-rated health, and fatigue or lethargy are included as disabilities in MFI, although they have not been reported to be associated with ALS previously. However, several epidemiological studies have found them as important features for the elderly population (26–28), further supporting the association between the physiological aging process and ALS risk.

In summary, the five indicators constituting the MFI fall into two categories. The first category includes nonspecific prodromal symptoms of neurodegenerative diseases, such as whole-body pain, fatigue, and poor self-rated health. The second category comprises indicators that characterize an individual’s frail state, such as falls and long-standing illness or infirmity. Notably, although the association between the MFI and ALS remained significant after excluding cases diagnosed within the first 8 years of follow-up, and time-dependent ROC analysis further revealed that Model B consistently achieved an AUC exceeding 0.7 throughout the first 10 years. But we ultimately position the MFI as a pragmatic early screening tool.

However, for a rare disease such as ALS, any screening approach will inevitably generate false-positive results. Even with the moderate C-index (C-index = 0.696) observed in our study, the psychological distress and healthcare burden arising from false positives cannot be overlooked. The MFI should therefore be regarded as a triage tool rather than a diagnostic instrument. Although biomarkers such as neurofilament light chain (NfL) exhibit stronger discriminatory performance (29–32), their current role is primarily confined to clinical diagnostic settings and they are impractical for community-based screening. As shown in Figure 3, individuals in the highest decile of the MFI-based risk score had a 15.84-fold higher ALS incidence compared with those in the lowest decile in the validation set. This suggests that the MFI could serve as a first-step screening tool, and that coupling it with biomarker testing, such as NfL, in high-scoring individuals may help improve early detection and shorten diagnostic delays.

### Strengths and limitations

We utilized a large sample of cohort data from the UK Biobank for our analysis, which well improves the robustness of our findings. The innovative use of deficits associated with ALS to calculate the MFI in our study improves the performance of the model.

However, there are limitations in our study. First, the various deficits reported by UK Biobank participants were collected in the form of self-administered questionnaires, which may be subject to reporting bias. But for potential non-differential misclassification, it would only underestimate the association between MFI and ALS, making the results more conservative. For differential misclassification, the effect remains significant even after excluding patients from the 8 years prior to cohort entry, indicating the robustness of the association between MFI and ALS. Second, only 49 deficits were examined in the UK Biobank database, which may miss certain deficits associated with ALS. Future studies incorporating more comprehensive clinical phenotypes may yield risk stratification tools with improved discriminatory performance. Third, despite the large cohort size of nearly 500,000 participants, only 628 incident ALS cases occurred during follow-up. The limited number of events may constrain statistical power and increase susceptibility to random variation, a challenge inherent to prospective research on rare diseases like ALS. Finally, our model was developed and validated exclusively within UK Biobank, a cohort that is healthier and more advantaged than the general population. External validation in more diverse populations is therefore needed.

## Conclusion

We constructed two risk stratification tools using demographic information and self-reported deficits from the UKB, and innovatively calculated the MFI. Through comparisons of the C-index and time-dependent ROC curves, it was found that the model by the combined use of MFI, gender, age and BMI showed the best discriminative ability. To a certain extent, this model can serve as a potential tool for identifying high-risk groups of ALS.

## Data Availability

The data analyzed in this study is subject to the following licenses/restrictions: UK Biobank data are available on request. Requests to access these datasets should be directed to https://www.ukbiobank.ac.uk/.
