# Supplementary material for: A modified frailty index to identify high-risk groups for amyotrophic lateral sclerosis
Source: Front Neurol. 2026 Jul 15;17:1879866. doi: 10.3389/fneur.2026.1879866 (PMC13414953; doi:10.3389/fneur.2026.1879866)
Supplement: Supplementary file 1 [file Table_1.DOCX]

Supplementary Table 1 Baseline characteristics of training and validation sets

| Characteristic | Training set (n = 350,024) | | |  | Validation set (n = 150,009) | | |
| --- | --- | --- | --- | --- | --- | --- | --- |
|  | Non-ALS | ALS | *P*-value |  | Non-ALS | ALS | *P*-value |
| Age (years,%) |  |  |  |  |  |  |  |
| < 60 | 198,425(99.92) | 157(0.08) | ＜0.001 |  | 84,782(99.93) | 59(0.07) | ＜0.001 |
| ≥ 60 | 151,169(99.82) | 273(0.18) |  |  | 65,029(99.81) | 139(0.21) |  |
| Gender (n,%) |  |  |  |  |  |  |  |
| Male | 159,207(99.85) | 246(0.15) | ＜0.001 |  | 68,347(99.85) | 106(0.15) | 0.025 |
| Female | 190,387(99.90) | 184(0.10) |  |  | 81,464(99.89) | 92(0.11) |  |
| Ethnicity (n,%) |  |  |  |  |  |  |  |
| White | 331,118(99.88) | 414(0.12) | 0.147 |  | 141,751(99.86) | 195(0.14) | 0.016 |
| Other | 18,476(99.95) | 16(0.09) |  |  | 8,060(99.94) | 3(0.04) |  |
| BMI (kg/m^2^,%) |  |  |  |  |  |  |  |
| < 25 | 114,960(99.88) | 140(0.18) | 0.886 |  | 49,262(99.86) | 67(0.14) | 0.775 |
| ≥ 25 | 234,634(99.88) | 290(0.12) |  |  | 100,549(99.87) | 131(0.13) |  |
| Smoking (n,%) |  |  |  |  |  |  |  |
| Never | 192,098(99.89) | 210(0.11) | 0.011 |  | 82,151(99.87) | 103(0.13) | 0.426 |
| Ever | 157,496(99.86) | 220(0.14) |  |  | 67,660(99.86) | 95(0.14) |  |
| Alcohol (n,%) |  |  |  |  |  |  |  |
| Never or occasional | 107,622(99.88) | 129(0.12) | 0.725 |  | 45,899(99.87) | 59(0.13) | 0.798 |
| Every week | 241,972(99.88) | 301(0.12) |  |  | 103,912(99.87) | 139(0.13) |  |
| TD index (n,%) |  |  |  |  |  |  |  |
| < -2.14 | 174,919(99.88) | 215(0.12) | 0.988 |  | 75,093(99.86) | 102(0.14) | 0.696 |
| ≥ -2.14 | 174,675(99.88) | 217(0.12) |  |  | 74,718(99.87) | 96(0.13) |  |

Supplementary Table 2. The associations of deficits with ALS

| deficit | HR（95%CI） | *P*-valve | Adjusted *P*-valve |
| --- | --- | --- | --- |
| Glaucoma | 0.71（0.31-1.58） | 0.398 | 0.929 |
| Cataracts | 0.74（0.43-1.27） | 0.280 | 0.762 |
| Hearing problems | 1.21（0.98-1.49） | 0.079 | 0.352 |
| Long-standing illness or infirmity | 1.42（1.16-1.73） | 0.001 | 0.012 |
| Self-rated health | 2.00（1.31-3.04） | 0.001 | 0.012 |
| Falls | 2.13（1.62-2.81） | ＜0.001 | 0.002 |
| Wheeze | 1.01（0.80-1.28） | 0.930 | 0.949 |
| Tiredness or lethargy | 1.67（1.21-2.31） | 0.002 | 0.020 |
| Myocardial infarction | 1.05（0.63-1.78） | 0.843 | 0.949 |
| Angina | 0.91（0.56-1.46） | 0.682 | 0.949 |
| Stroke | 1.47（0.83-2.63） | 0.188 | 0.658 |
| Hypertension | 1.04（0.84-1.88） | 0.722 | 0.949 |
| Rheumatoid arthritis | 0.77（0.29-2.07） | 0.604 | 0.949 |
| Osteoarthritis | 1.09（0.80-1.50） | 0.586 | 0.949 |
| Gout | 0.73（0.33-1.64） | 0.449 | 0.949 |
| Dental problems | 1.30（1.07-1.58） | 0.007 | 0.057 |
| Chest pain | 0.98（0.76-1.28） | 0.898 | 0.949 |
| Sciatica | 1.19（0.50-2.89） | 0.689 | 0.949 |
| Diabetes | 1.26（0.88-1.81） | 0.215 | 0.662 |
| Cancer | 1.18（0.86-1.61） | 0.305 | 0.787 |
| Multiple cancers | 0.81（0.20-3.23） | 0.761 | 0.949 |
| Fractures | 1.02（0.74-1.41） | 0.911 | 0.949 |
| Deep vein thrombosis | 1.03（0.55-1.93） | 0.923 | 0.949 |
| Emphysema | 0.82（0.39-1.73） | 0.604 | 0.949 |
| Asthma | 0.98（0.73-1.33） | 0.921 | 0.949 |
| Allergies | 1.03（0.82-1.30） | 0.787 | 0.949 |
| Hypothyroidism | 0.73（0.44-1.24） | 0.244 | 0.703 |
| Depression | 1.37（0.94-2.00） | 0.099 | 0.404 |
| Anxiousness | 0.86（0.68-1.09） | 0.216 | 0.662 |
| Severe anxiety | 1.15（0.51-2.58） | 0.732 | 0.949 |
| Misery | 1.03（0.85-1.26） | 0.744 | 0.949 |
| Loneliness | 1.28（1.01-1.63） | 0.041 | 0.251 |
| Head and/or neck pain | 0.92（0.75-1.13） | 0.430 | 0.949 |
| Back pain | 1.17（0.95-1.44） | 0.137 | 0.516 |
| Stomach/abdominal pain | 1.13（0.80-1.58） | 0.487 | 0.949 |
| Hip pain | 1.28（0.98-1.68） | 0.071 | 0.348 |
| Knee pain | 0.89（0.70-1.13） | 0.331 | 0.811 |
| Whole-body pain | 2.64（1.64-4.25） | ＜0.001 | 0.002 |
| Facial pain | 0.99（0.47-2.10） | 0.987 | 0.987 |
| Sleep | 1.10（0.85-1.44） | 0.468 | 0.949 |
| High cholesterol | 0.75（0.59-0.97） | 0.028 | 0.196 |
| Pneumonia | 0.89（0.40-2.00） | 0.782 | 0.949 |
| Gastric reflux | 1.11（0.72-1.71） | 0.622 | 0.949 |
| Hiatus hernia | 0.81（0.42-1.57） | 0.531 | 0.949 |
| Diverticulitis | 1.08（0.48-2.42） | 0.855 | 0.949 |
| Gall stones | 1.12（0.55-2.26） | 0.752 | 0.949 |
| Psoriasis | 1.85（0.95-3.58） | 0.069 | 0.348 |
| Osteoporosis | 0.79（0.35-1.78） | 0.570 | 0.949 |
| Migraine | 1.06（0.58-1.94） | 0.839 | 0.949 |

Supplementary Table 3. Variables and AUC of the different risk prediction models

| Model | Variable | Multivariate Cox | | | |  | |
| --- | --- | --- | --- | --- | --- | --- | --- |
|  |  | *β* | HR (95%CI) | *P*-valve | training set C-index | validation set C-index | |
| Model A | FI | 1.53 | 4.60 (1.38~15.36) | 0.013 | 0.661 (0.637~0.686) | | 0.688 (0.654~0.722) |
|  | Gender | 0.48 | 1.62 (1.34~1.96) | < 0.001 |  |  |  |
|  | Age | 0.07 | 1.07 (1.05~1.08) | < 0.001 |  |  |  |
| Model B | MFI | 1.54 | 4.66 (2.88~7.53) | < 0.001 | 0.676 (0.652~0.701) | 0.696 (0.663~0.730) | |
|  | Gender | 0.51 | 1.66 (1.37~2.01) | < 0.001 |  |  |  |
|  | Age | 0.07 | 1.07 (1.06~1.09) | < 0.001 |  |  |  |
|  | BMI | -0.21 | 0.79 (0.66~0.99) | 0.043 |  |  |  |

Model A: FI based model incorporating gender and age.

Model B: MFI based model incorporating gender,age and BMI.


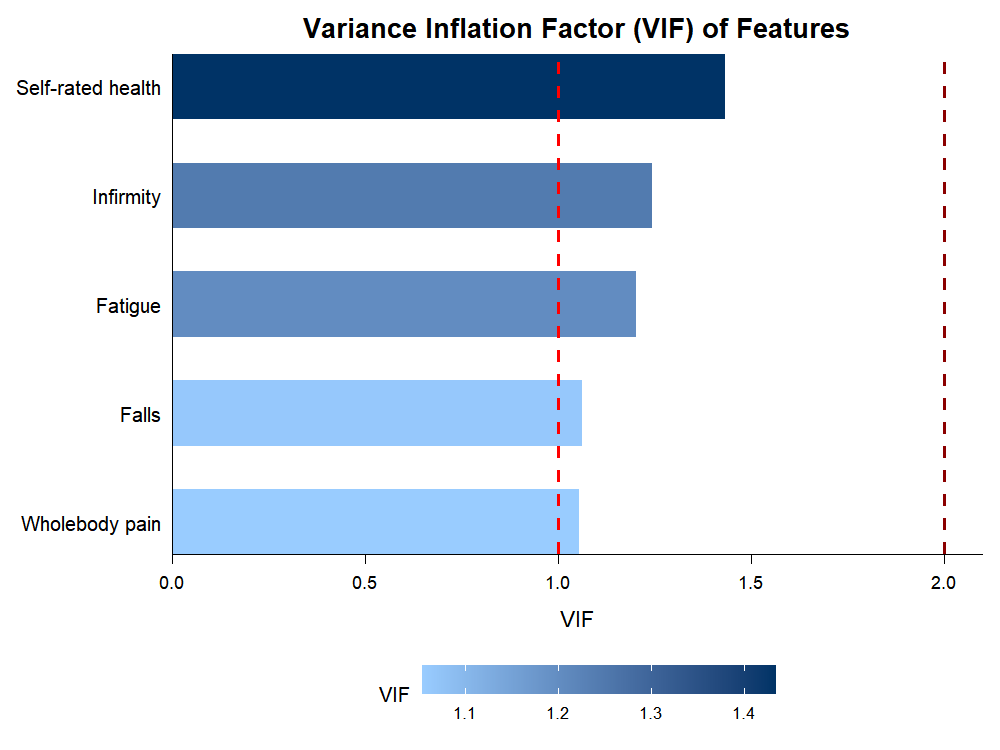


**Supplementary Figure 1.** Variance Inflation Factor (VIF) of Features


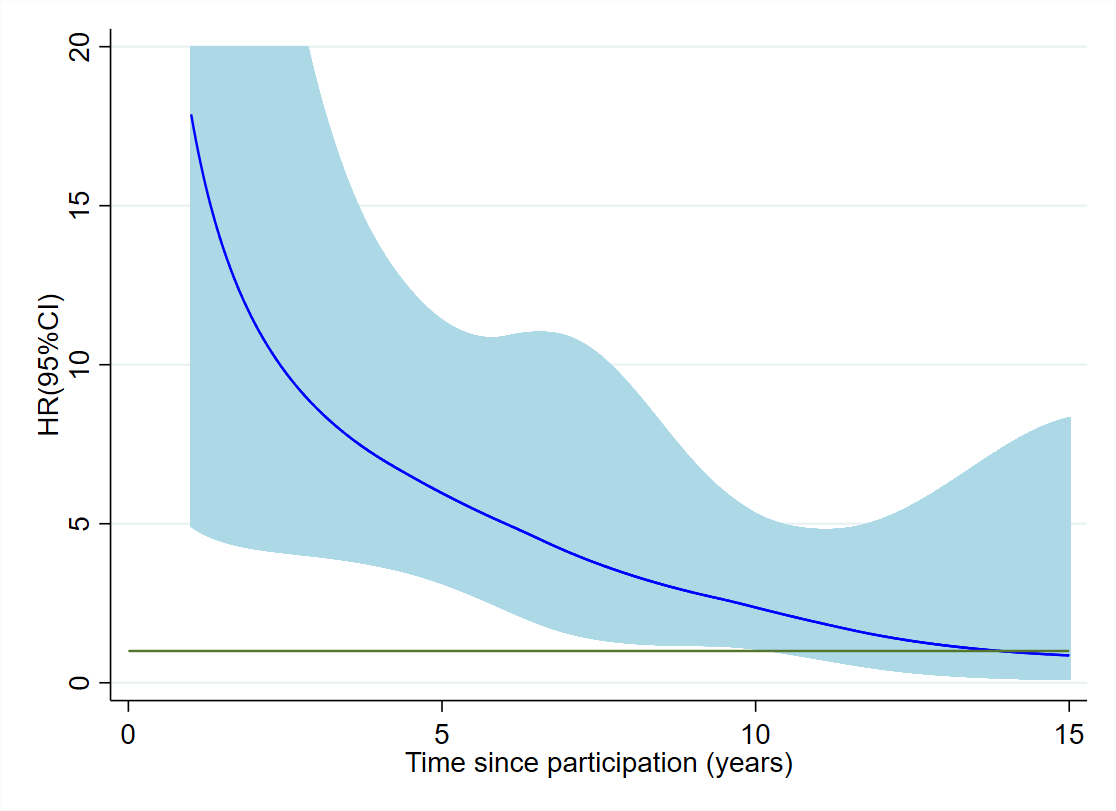


**Supplementary Figure 2.** Plot of the time dependent HR for the MFI.
